# Development and validation of an individualized nomogram to identify occult peritoneal metastasis in patients with advanced gastric cancer

**DOI:** 10.1093/annonc/mdz001

**Published:** 2019-01-23

**Authors:** D Dong, L Tang, Z -Y Li, M -J Fang, J -B Gao, X -H Shan, X -J Ying, Y -S Sun, J Fu, X -X Wang, L -M Li, Z -H Li, D -F Zhang, Y Zhang, Z -M Li, F Shan, Z -D Bu, J Tian, J -F Ji

**Affiliations:** 1 CAS Key Laboratory of Molecular Imaging, Institute of Automation, Chinese Academy of Sciences, Beijing; 2 Key Laboratory of Carcinogenesis and Translational Research (Ministry of Education), Radiology Department, , Peking University Cancer Hospital & Institute, Beijing; 3 University of Chinese Academy of Sciences, Beijing; 4 Key Laboratory of Carcinogenesis and Translational Research (Ministry of Education), Gastrointestinal Cancer Center, Peking University Cancer Hospital & Institute, Beijing; 5 Department of Radiology, The First Affiliated Hospital of Zhengzhou University, Zhengzhou; 6 Department of Radiology, Affiliated People’s Hospital of Jiangsu University, Zhenjiang; 7 Department of Radiology, The Third Affiliated Hospital of Kunming Medical University, Yunnan Cancer Hospital, Kunming; 8 Beijing Advanced Innovation Center for Big Data-Based Precision Medicine, School of Medicine, Beihang University, Beijing, China

**Keywords:** occult peritoneal metastasis, radiomic nomogram, advanced gastric cancer

## Abstract

**Background:**

Occult peritoneal metastasis (PM) in advanced gastric cancer (AGC) patients is highly possible to be missed on computed tomography (CT) images. Patients with occult PMs are subject to late detection or even improper surgical treatment. We therefore aimed to develop a radiomic nomogram to preoperatively identify occult PMs in AGC patients.

**Patients and methods:**

A total of 554 AGC patients from 4 centers were divided into 1 training, 1 internal validation, and 2 external validation cohorts. All patients’ PM status was firstly diagnosed as negative by CT, but later confirmed by laparoscopy (PM-positive *n *=* *122, PM-negative *n *=* *432). Radiomic signatures reflecting phenotypes of the primary tumor (RS1) and peritoneum region (RS2) were built as predictors of PM from 266 quantitative image features. Individualized nomograms of PM status incorporating RS1, RS2, or clinical factors were developed and evaluated regarding prediction ability.

**Results:**

RS1, RS2, and Lauren type were significant predictors of occult PM (all *P *<* *0.05). A nomogram of these three factors demonstrated better diagnostic accuracy than the model with RS1, RS2, or clinical factors alone (all net reclassification improvement *P *<* *0.05). The area under curve yielded was 0.958 [95% confidence interval (CI) 0.923–0.993], 0.941 (95% CI 0.904–0.977), 0.928 (95% CI 0.886–0.971), and 0.920 (95% CI 0.862–0.978) for the training, internal, and two external validation cohorts, respectively. Stratification analysis showed that this nomogram had potential generalization ability.

**Conclusion:**

CT phenotypes of both primary tumor and nearby peritoneum are significantly associated with occult PM status. A nomogram of these CT phenotypes and Lauren type has an excellent prediction ability of occult PM, and may have significant clinical implications on early detection of occult PM for AGC.

**Key Message**

Computed tomography radiomic signatures reflecting phenotypes of primary tumor and nearby peritoneum, as well as the Lauren type were significant predictors for occult peritoneal metastasis (PM) in advanced gastric cancer (AGC) patients. A radiomic nomogram, combining these predictors, could preoperatively identify occult PM, and thereby facilitate the individual treatment of AGC.

## Introduction

Peritoneal metastasis (PM) occurs in ∼53%–66% of patients with distant metastatic gastric cancer [1, 2]. Early detection and diagnosis of PM is clinically significant regarding optimal treatment selection and avoidance of unnecessary surgical procedures. 

Computed tomography (CT) is the most common noninvasive modality to diagnose PM [3]. The conventional CT indications for PM include omental cake, large amount of ascites, and obvious parietal peritoneum thickening [4]. However, most of these signs usually appear in late-stage PM. CT detection of PM is therefore believed to have high specificity but low sensitivity (∼50%) [5]. This raises a problem in clinical practice: ∼10%–30% of CT-diagnosed PM-negative advanced gastric cancer (AGC) patients were confirmed as PM-positive during subsequent laparoscopies, named occult PM [4, 5]. Even with a multidisciplinary discussion, ∼16.7% of PMs are undetected [6].

Both European Society for Medical Oncology (ESMO) [3] and National Comprehensive Cancer Network (NCCN) [6] guidelines recommended that laparoscopy exploration should be applied to patients with potentially resectable AGC to detect occult PM. However, because laparoscopy is an invasive diagnostic procedure, selection of patients appropriate for laparoscopy exploration is still controversial. Studies have been conducted to identify risk factors associated with PM among gastric cancer patients, including TN staging, Borrmann classifications, and entropy of the omentum [4, 7–9], however, by far no individualized prediction model has been developed.

Radiomics is a novel tool which extracts hundreds of quantitative features from medical imaging, and combines key features into an image-based biomarker (named radiomic signature) for cancer diagnostics [10, 11]. There have been several applications of radiomics in gastro-intestinal tumors, such as response assessment of neoadjuvant chemoradiation in rectal cancer [12], prediction of lymph node metastasis in colorectal cancer [13], and differentiation of tumor types in gastric cancer [14]. These studies highlight the value of radiomics, which can also be a potential tool for decoding atypical indications of occult PM on CT imaging. We therefore developed and validated a radiomic model for noninvasive prediction of PM status in AGC preoperatively.

## Patients and methods

### Patients

Upon attaining the ethical approval from the institutional review board in all participating centers, 554 patients were retrospectively selected. The inclusion/exclusion criteria and patient recruitment process are shown in supplementary A1, available at *Annals of Oncology* online. The need for informed patient consent was waived.

All included patients were initially diagnosed as PM-negative by CT, but later confirmed with the actual PM status in laparoscopic exploration. One hundred and twenty-two patients had occult PM-positive status and 432 had true PM-negative status. As shown in supplementary A1 and Figure S1, available at *Annals of Oncology* online, the patients were divided into four cohorts: one training cohort (*n *=* *100 from center 1), one internal validation cohort (*n *=* *226 from center 1), and two external validation cohorts (*n *=* *131 from center 2 and center 3, *n *=* *97 from center 4). The sample size consideration is shown in supplementary A2, available at *Annals of Oncology* online.

### CT examination 

All patients underwent enhanced CT examination within two weeks before laparoscopy. The details of the CT protocol are shown in supplementary A3 and Table S1, available at *Annals of Oncology* online.

**Table 1.** Characteristics of patients in the training and validation cohorts

Characteristic	Training cohort			Internal-validation cohort			External-validation cohort 1			External-validation cohort 2		
	PM (+)	PM (−)	*P* value	PM (+)	PM (−)	*P* value	PM (+)	PM (−)	*P* value	PM (+)	PM (−)	*P* value
Sex, No. (%)			0.0897*			0.1698			0.0022*			0.4991
Male	35 (70.0)	43 (86.0)		12 (60.0)	153 (74.3)		13 (48.1)	81 (77.9)		16 (66.7)	43 (58.9)	
Female	15 (30.0)	7 (14.0)		8 (40.0)	53 (25.7)		14 (51.9)	23 (22.1)		8 (33.3)	30 (41.1)	
Age, mean ± SD, years	59.76 ± 12.71	58.12 ± 10.32	0.3446	57.30 ± 13.63	60.34 ± 11.06	0.3121	55.74 ± 12.82	58.54 ± 10.33	0.2155	61.08 ± 11.59	61.74 ± 9.56	0.9933
Mild-CT-defined ascites, No. (%)			0.0040*			0.0075*			0.1918			–
+	10 (20.0)	1 (2.0)		2 (10.0)	0 (0.0)		3 (11.1)	3 (2.9)		–	–	
−	40 (80.0)	49 (98.0)		18 (90.0)	206 (100.0)		24 (88.9)	101 (97.1)		–	–	
Locations, No. (%)			0.0071*			0.0002*			0.3251			0.2997
Gastric antrum	20 (40.0)	14 (28.0)		8 (40.0)	47 (22.8)		10 (37.0)	29 (27.9)		6 (37.5)	18 (24.7)	
Gastric body	15 (30.0)	16 (32.0)		4 (20.0)	66 (32.0)		7 (25.9)	32 (30.8)		2 (12.5)	27 (37.0)	
Esophagogastric junction	7 (14.0)	19 (38.0)		4 (20.0)	91 (44.2)		6 (22.2)	36 (34.6)		3 (18.8)	11 (15.1)	
Whole stomach	8 (16.0)	1 (2.0)		4 (20.0)	2 (1.0)		4 (14.8)	7 (6.7)		5 (31.3)	17 (23.3)	
Pathology, No. (%)			0.0838*			0.9875			0.0271*			0.7199
Adenocarcinoma	40 (80.0)	46 (92.0)		17 (85.0)	181 (87.9)		21 (77.8)	97 (93.3)		14 (93.3)	66 (90.4)	
Signet ring and mucinous cell carcinoma	10 (20.0)	4 (8.0)		3 (15.0)	25 (12.1)		6 (22.2)	7 (6.7)		1 (6.7)	7 (9.6)	
Differentiation, No. (%)			0.8412			0.0373*			0.0002*			0.2959
Poorly differentiated	27 (54.0)	26 (52.0)		14 (70.0)	93 (45.1)		21 (77.8)	38 (36.5)		11 (73.3)	43 (58.9)	
Moderately and well differentiated	23 (46.0)	24 (48.0)		6 (30.0)	113 (54.9)		6 (22.2)	66 (63.5)		4 (26.7)	30 (41.1)	
Lauren type, No. (%)			0.0019*			0.0149*			0.0846*			–
Intestinal type and mixed type	24 (48.0)	39 (78.0)		9 (45.0)	147 (31.6)		10 (37.0)	59 (56.7)		–	–	
Diffuse type	26 (52.0)	11 (22.0)		11 (55.0)	59 (28.6)		17 (63.0)	45 (43.3)		–	–	
Borrmann type, No. (%)			0.0158*			0.0002*			0.0026*			–
Types 2 and 3	34 (68.0)	44 (88.0)		15 (75.0)	203 (98.5)		20 (74.1)	99 (95.2)		–	–	
Type 4	16 (32.0)	6 (12.0)		5 (25.0)	3 (1.5)		7 (25.9)	5 (4.8)		–	–	
CEA, No. (%)			0.3618			0.4361			0.8873			0.6039
Normal	35 (70.0)	39 (78.0)		18 (90.0)	165 (80.1)		25 (92.6)	89 (91.8)		9 (69.2)	53 (80.3)	
Elevated	15 (30.0)	11 (22.0)		2 (10.0)	41 (19.9)		2 (7.4)	8 (8.2)		4 (30.8)	13 (19.7)	
CA19-9, No. (%)			0.1059			0.7711			0.8902			0.4543
Normal	34 (68.0)	41 (82.0)		16 (80.0)	168 (81.6)		23 (85.2)	85 (88.5)		12 (92.3)	48 (78.7)	
Elevated	16 (32.0)	9 (18.0)		4 (20.0)	38 (18.4)		4 (14.8)	11 (11.5)		1 (8.7)	13 (21.3)	

*P* value was derived from the univariable association analyses between each characteristic and PM status.

SD, standard deviation; CEA, carcinoembryonic antigen; CA19-9, carbohydrate antigen 19-9.

* *P* value < 0.1.

### PM status ascertainment

All patients underwent diagnostic laparoscopy. Any suspicious lesion discovered during laparoscopy was biopsied and pathologically examined to determine PM status. Detailed description of the laparoscopy procedure is shown in supplementary A4, available at *Annals of Oncology* online.

### Radiomic signature building

**Figure 1.**
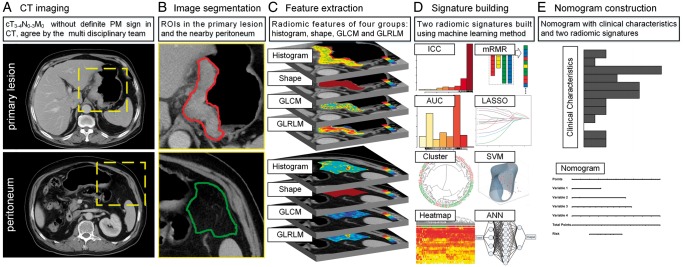
Radiomics workflow in this study. During the image segmentation, any detectable large blood vessels were excluded from the ROIs.

Figure 1 shows the workflow of this study. Venous phase CT images were retrieved from our Picture Archiving and Communication System and then exported to the ITK-SNAP software (version 2.2.0; www.itksnap.org) for manual segmentation. Considering that PM initiation depends on the synergies of the primary tumor and peritoneal microenvironment [15], both of their characteristics under CT scanning are investigated. For the primary tumor, radiologists reviewed all slices of a patient and selected one slice with the largest tumor area. A 2D region of interest (ROI-1) of the tumor was then delineated on this slice. For the peritoneum, radiologists selected one slice with the peritoneal region (area > 2 cm^2^) nearest to the center of the primary tumor. ROI-2 was delineated on this slice to cover the peritoneal region.

Two groups of features (133 features each) were extracted from ROI-1 and ROI-2. These features included histogram, shape, gray-level co-occurrence matrix (GLCM), and gray-level run-length matrix (GLRLM) (supplementary A5, available at *Annals of Oncology* online). Unsupervised clustering and radiomic heatmaps were used to reveal patient clusters of similar radiomic-expression patterns and their associations with PM. As shown in supplementary A6, available at *Annals of Oncology* online, feature selection and signature building process were carried out on both primary tumor and peritoneum including three steps: (i) feature reproducibility assessment on inter-/intra-reader agreement and slice-thickness agreement; (ii) reservation of top ranking features with mutual information; (iii) signature building with comparison among three state-of-the-art methods. After these steps, a radiomic signature reflecting the features of the primary tumor (RS1) and another radiomic signature reflecting that of peritoneum (RS2) were built as predictors of PM status.

### Radiomic nomogram construction

Univariate analysis was used to assess the association between clinical characteristics and PM. Differences in patient characteristics by PM status were assessed using the independent *T*-test or Mann–Whitney *U* test for continuous variables, and Fisher’s exact test or *χ*^2^ test for categorical variables.

Multivariable logistic regression was applied to select independent predictors of PM from the radiomic signatures and significant clinical characteristics. We built a radiomic nomogram with both radiomic and clinical features, as well as a clinical model containing only the clinical characteristics for comparison.

### Radiomic nomogram evaluation

The accuracy of the radiomic nomogram was assessed with the receiver-operating characteristic (ROC) curve. The area under ROC curve (AUC) was calculated and compared between training and validation cohorts using the DeLong test. Sensitivity and specificity were also calculated. The calibration of the radiomic signatures and nomogram were assessed using the calibration curves and Hosmer–Lemeshow test. Considering the group imbalance in the validation cohorts, we carried out 1000 bootstrapping resamples in each group for internal and external validation.

Net reclassification index (NRI) was calculated to compare the performance between radiomic nomogram and clinical model. Moreover, we carried out stratification analysis on patient characteristics and CT protocol. Decision curve analysis was conducted to evaluate the radiomic nomogram’s clinical usefulness by quantifying the net benefit at different threshold probabilities.

### Statistical analysis

Statistical analysis was conducted with R software (version 3.5.0; http://www.Rproject.org) and MATLAB (version 2017a; Mathworks, Natick, MA). A two-sided *P* value <0.05 was used to indicate statistical significance. 

## Results

### Clinical characteristics

Clinical characteristics, including mild CT-defined ascites (difficult to determine benign or malignant by radiologists), tumor locations, Lauren type, and Borrmann type were significantly associated with PM after univariate analysis (*P *<* *0.05; Table 1).

### Feature selection and radiomic signature building

After assessing reproducibility, 93 features from primary tumor (ROI-1) and 98 features from the peritoneum (ROI-2) were selected. The heatmaps of these features and unsupervised cluster partitioning are shown in supplementary Figure S2, available at *Annals of Oncology* online. A significant association between these features and PM was observed. After ranking these features, the top 20 features from primary tumor and the top 20 features from peritoneum were selected.

We compared three methods for signature building and found the Least Absolute Shrinkage and Selection Operator Method (LASSO) logistic regression model carried out the best (see supplementary Table S2, available at *Annals of Oncology* online). As shown in supplementary A7, Figure S3, and Table S3, available at *Annals of Oncology* online, LASSO selected two-key features from primary tumor and two-key features from peritoneum into two radiomic signatures, RS1 (XO_H_mass and XH_GLRLM_entropy) and RS2 (XL_H_energy and XL_GLCM_entropy). The radiomic signatures yielded significant difference in value between PM-positive and PM-negative groups (independent *T*-test *P *<* *0.0001 in all cases). The feature maps of one PM-negative and one PM-positive patient are shown in supplementary Figure S4, available at *Annals of Oncology* online. Because RS1 and RS2 were extracted from single CT slices, they might be affected by slice selection from the radiologists. We tested the consistency of radiomic signatures among the slice selection of ROI-1 and ROI-2 from a random sample of 30 cases and found that the two signatures had a good consistency among the slice selections (supplementary A8, available at *Annals of Oncology* online).

**Table 2.** Variables and coefficients of radiomic nomogram and clinical model

Variable	Radiomic nomogram			Clinical model		
	β	Adjusted OR (95% CI)	*P* value	β	Adjusted OR (95% CI)	*P* value
Intercept	−6.972			−1.051		
Mild CT-defined ascites (+ versus −)	–	–	–	2.448	11.560 (1.321–101.170)	0.0270
Lauren (diffuse versus intestinal/mixed)	2.704	14.939 (2.091–106.720)	0.0070	1.312	3.713 (1.435–9.611)	0.0069
Borrmann (type 4 versus type 2/type 3)	–	–	–	1.262	3.532 (1.150–10.848)	0.0275
RS1 score (per 0.1 increase)	0.489	1.630 (1.224–2.172)	0.0008	–	–	–
RS2 score (per 0.1 increase)	0.739	2.094 (1.509–2.905)	<0.0001	–	–	–

RS, radiomic signature; OR, odds ratio; CI, confidence interval.

**Table 3.** Performance evaluation of the radiomic models

Index	Training cohort				Internal-validation cohort			
	RS1	RS2	Clinical model	Nomogram	RS1	RS2	Clinical model	Nomogram
TP	36	38	19	45	17	15	4	17
TN	40	41	49	41	157	179	204	179
FN	14	12	31	5	3	5	16	3
FP	10	9	1	9	49	27	2	27
Sensitivity	0.720	0.760	0.380	0.900	0.850	0.750	0.200	0.850
Specificity	0.800	0.820	0.980	0.820	0.762	0.869	0.990	0.869
AUC	0.854	0.906	0.694	0.958	0.868	0.873	0.650	0.941
	(0.781–0.926)	(0.850–0.961)	(0.598–0.790)	(0.923–0.993)	(0.800–0.936)	(0.7750.970)	(0.523–0.777)	(0.904–0.977)

Index	External-validation cohort 1				External-validation cohort 2			
	RS1	RS2	Clinical model	Nomogram	RS1	RS2	Clinical model	Nomogram
TP	19	21	7	25	16	17	–	17
TN	85	84	97	86	59	65	–	66
FN	8	6	20	2	8	7	–	7
FP	19	20	7	18	14	8	–	7
Sensitivity	0.704	0.778	0.259	0.926	0.667	0.708	–	0.708
Specificity	0.817	0.808	0.933	0.827	0.808	0.890	–	0.904
AUC	0.894	0.849	0.675	0.928	0.828	0.870	–	0.920
	(0.836–0.953)	(0.755–0.943)	(0.566–0.783)	(0.886–0.971)	(0.742–0.915)	(0.776–0.965)		(0.862–0.978)

TP, true positive; TN, true negative; FN, false negative; FP, false positive; AUC, area under curve; CI, confidence interval; RS1, radiomic signature from the primary tumor; RS2, radiomic signature from the peritoneal region.

### Radiomic nomogram construction and validation

**Figure 2.**
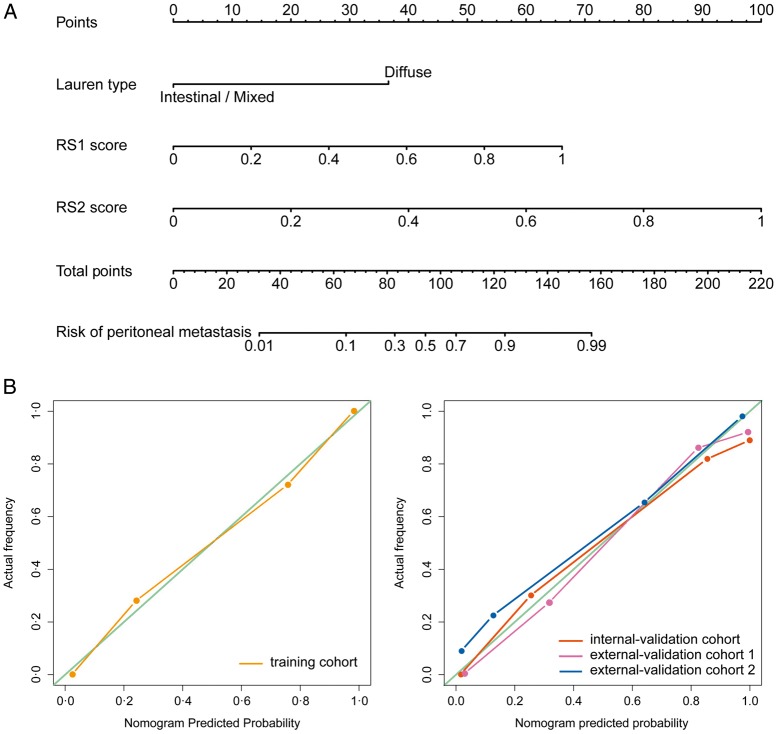
Development and performance of nomogram. (A) Nomogram based on radiomic signatures and clinical factors. Calibration curves of the radiomic nomogram in the training cohort (B) and validation cohorts (C).

Multivariable analysis of clinical characteristics and radiomic signatures revealed that Lauren type, RS1, and RS2 were significant predictors (Table 2). Therefore, they were fused as a radiomic nomogram (Figure 2A). There were significant differences in the nomogram predicted value between PM-positive and PM-negative groups in each cohort (independent *T*-test *P *<* *0.0001, Table 3). Considering that Lauren type determined from the endoscopic biopsy tissue might be lacked in other hospitals, a comparative model with only the two radiomic signatures was also built and validated on the external validation cohort number 2.

The NRI revealed that the nomogram had better predictive performance than the clinical-characteristics-only model (incorporating mild CT-defined ascites, Lauren type, and Borrmann type) in the internal validation cohort (NRI = 0.460, *P *=* *0.0008) and external validation cohort number 1 (NRI = 0.454, *P *<* *0.0001). In the bootstrapping validation, the nomogram also yielded high AUCs of 0.936 (95% CI 0.926–0.946), 0.925 (95% CI 0.913–0.937), and 0.917 (95% CI 0.906–0.928) in the internal validation cohort and two external validation cohorts, respectively. To assess possible overfitting, the Delong test was implemented on the ROC curves of the nomogram and revealed that the differences were not statistically significant among the AUCs of the training cohort and the three validation cohorts, with *P* values of 0.4995, 0.2948, and 0.2755, respectively.

The nomogram calibration curve demonstrated good agreement between prediction and observation in all cohorts (Figure 2B and C). The Hosmer–Lemeshow test was not significant (*P *>* *0.05), demonstrating a good fit.

As shown in supplementary A9, Figures S5 and S6, available at *Annals of Oncology* online, the stratified analysis showed that the performance of radiomic nomogram was not affected by patient sex, age, BMI, the version of CT, type of CT contrast agent, contrast agent concentration, contrast agent infused rate, or image thickness (Delong test *P *>* *0.05).

### Clinical use

**Figure 3.**
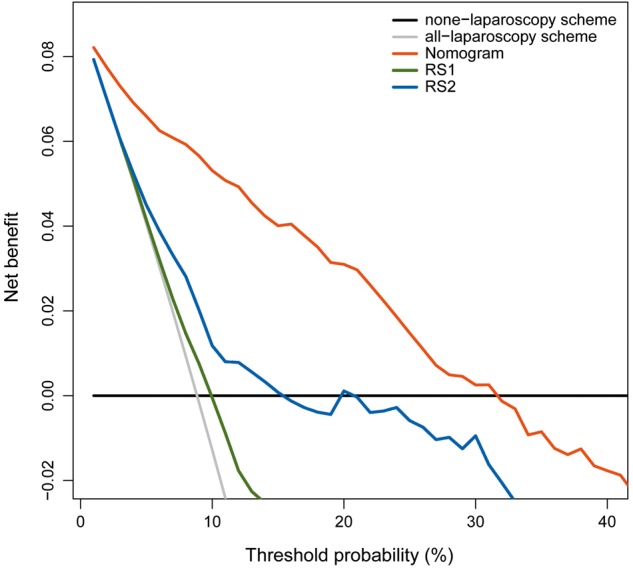
Decision curve analysis for radiomic nomogram and signatures.

The decision curve was used to compare the benefit of the radiomic nomogram, all-laparoscopy, and none-laparoscopy schemes. We found that if the threshold probability in clinical decision was <30% (i.e. if the improper surgical procedure for PM-positive patient was considered more harmful than laparoscopy exploration), the patients would benefit more from the nomogram than either of the all-laparoscopy or none-laparoscopy schemes (Figure 3). Moreover, the benefit of radiomic nomogram on the internal validation cohort was shown in supplementary A10, available at *Annals of Oncology* online.

## Discussion

In this study, we built a radiomic nomogram to identify occult PM using a relatively large datasets from four centers. The constructed nomogram provided an easy-to-use, preoperative, and individualized tool for PM diagnosis, which can help avoid improper surgical procedures for occult PM-positive patients or determine optimal candidates for laparoscopy exploration. In order to facilitate the usage of our nomogram, we made our nomogram open access in our website (www.radiomics.net.cn/platform.html).

The nearby peritoneum is the mostly probable position of distant metastasis in gastric cancer [2]. The ‘seed and soil’ theory (see Figure S7) proposes that PM initiation depends on the synergies of the tumor cells (seed) and the peritoneal microenvironment (soil) [15]. We believe that occult PM might be early-stage PM without typical CT signs. Interestingly, we found that occult PM was associated with not only the tumor itself, but also the phenotype of its nearby peritoneum. It is considered that tumor cells tend to deposit at lymphatic stomata or milky-spots and proliferate in the submesothelial space [16]. The findings may reflect the early process of the PM formation. We further analyzed the radiomic features in the nomogram (supplementary Figure S4, available at *Annals of Oncology* online). High ‘XH_GLRLM_entropy’ of primary tumor was correlated with high possibility of PM. We hypothesize that this nonuniform intensity distribution of the run length reflects the heterogeneity of the tumor: the more complex this intensity pattern is, the higher the heterogeneity and invasiveness of the tumor are, and thus the higher possibility of PM is. The feature ‘XO_H_mass’, which tends to emphasize the large tumor with high intensity level, may provide the information about the stage of tumor and yielded a high diagnostic significance for PM. Furthermore, two features reflecting the heterogeneity of the peritoneum were used, including ‘XL_H_energy’ and ‘XL_GLCM_entropy’. A peritoneum with high ‘GLCM_entropy’, reflecting low uniformity and high heterogeneity, was found to be sensitive to tumor metastasis. We hypothesize these features reflect the preclinical change of the peritoneal microenvironment, the proliferation of the free cancer cells in the area of ‘milky-spots’ [17], and the angiogenesis driven by vascular endothelial growth factor in the peritoneum, which may explain the heterogeneous change of peritoneal area.

Borrmann type, which reflects the aggressive biological behavior of tumor [18], was found significantly associated with PM in this study. One meta-analysis indicated that Borrmann type 4 gastric cancer was associated with higher possibility of PM [19]. Huang et al. [7] pointed that the odds of PM with Borrmann types 3–4 was 2.06 times that of those with Borrmann types 1–2. Hur et al. [8] suggested that Borrmann types 3–4 patients should undergo laparoscopy. In our study, we found Borrmann types had a strong correlation with RS1 (*P *<* *0.0001, Spearman correlation analysis with a permutation test) but lower weighted coefficient than RS1. Therefore, Borrmann type was replaced by more predictive radiomic features during the nomogram building.

Serum CEA and CA19-9 levels were likewise not in the nomogram. A previous study suggested that CEA could be a predictor for PM [20]. However, no statistical difference in CEA between PM was found in our univariate analysis. A possible reason for this finding was that only early-stage PM patients were enrolled in this study, of whom the serum indicators might not yet show its clinical significance and be particularly useful. This may also be the reason why pathological differentiation was noninformative in our study as well.

Our study has several limitations. The ROIs were delineated in one single slice (2D), which might not be representative of the entire tumor or peritoneum. Meanwhile, some radiomic features may be affected when extracted from 2D versus 3D images, particularly the texture features. Therefore, 3D analysis of the entire tumor or peritoneum should be further investigated. Moreover, the Lauren type in our nomogram was determined from the endoscopic biopsy specimen, but there may be minor discordance of Lauren classification between biopsy and surgical specimen. This discordance should also be studied. Furthermore, we used the retrospective datasets to develop the nomogram, of which some clinical factors such as CA125, HER-2 were not initially available and the accuracy of the information can be questionable.

In summary, a radiomic nomogram based on CT phenotypes and Lauren type was built for prediction of occult PM. The proposed nomogram is of great application potential in clinical practice in terms of individual treatment of AGC.

## Supplementary Material

Supplementary Data
