# Supplementary material for: Development and validation of an individualized nomogram to identify occult peritoneal metastasis in patients with advanced gastric cancer
Source: Ann Oncol. 2019 Jan 23;30(3):431–8. doi: 10.1093/annonc/mdz001 (PMC6442651; doi:10.1093/annonc/mdz001)
Supplement: Supplementary Data [file mdz001_supp.zip › mdz001-suppl_data/mdz001_Supplementary_Figure_S3.docx]

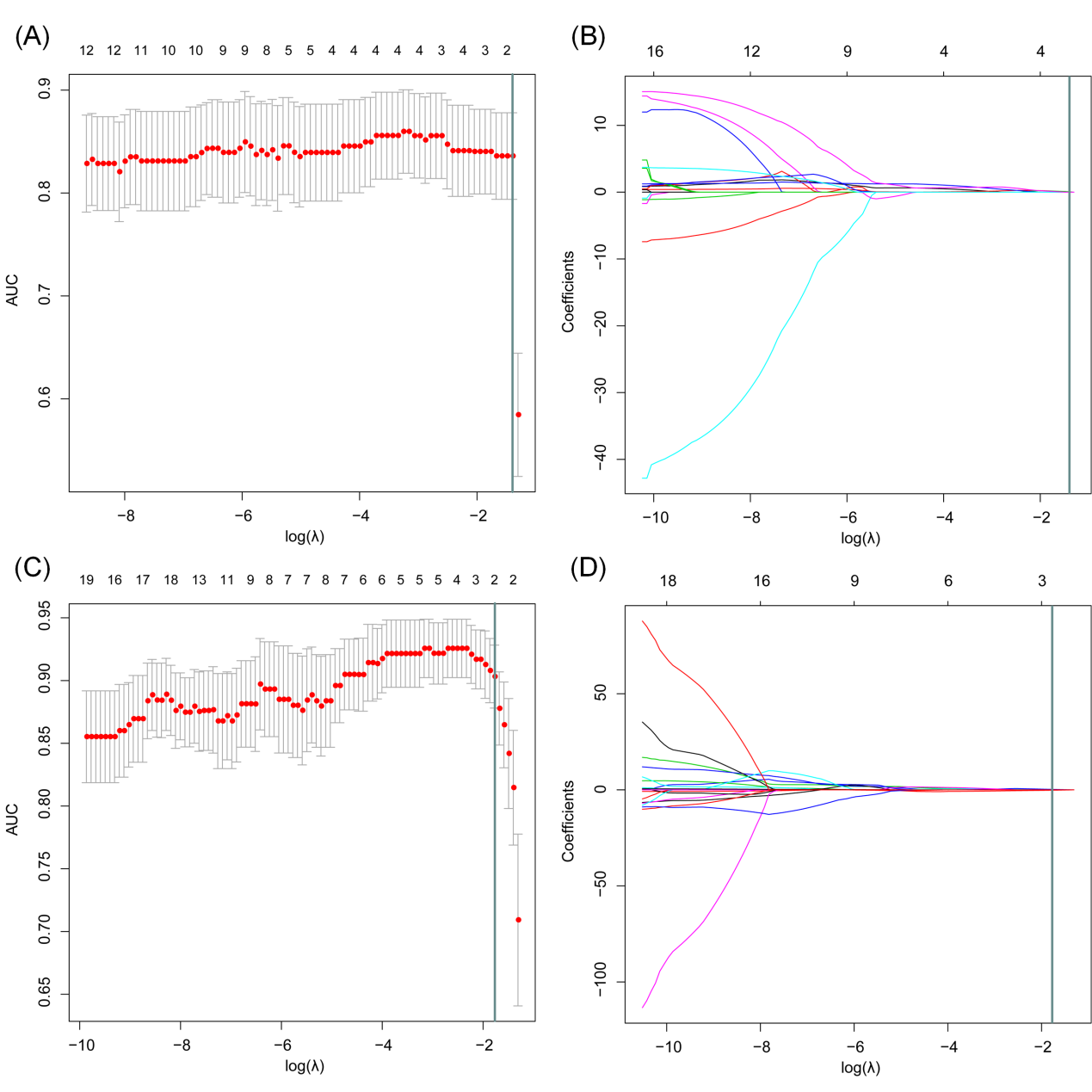


**Supplementary Figure S3**. LASSO logistic regression on features from the primary tumor (A, B) and peritoneum (C, D). The LASSO regression can reduce the feature dimension by shrinking the coefficients of some features to zero and further select key features.
