# Supplementary material for: Development and validation of an individualized nomogram to identify occult peritoneal metastasis in patients with advanced gastric cancer
Source: Ann Oncol. 2019 Jan 23;30(3):431–8. doi: 10.1093/annonc/mdz001 (PMC6442651; doi:10.1093/annonc/mdz001)
Supplement: Supplementary Data [file mdz001_supp.zip › mdz001-suppl_data/mdz001_Supplementary_Figure_S2.docx]

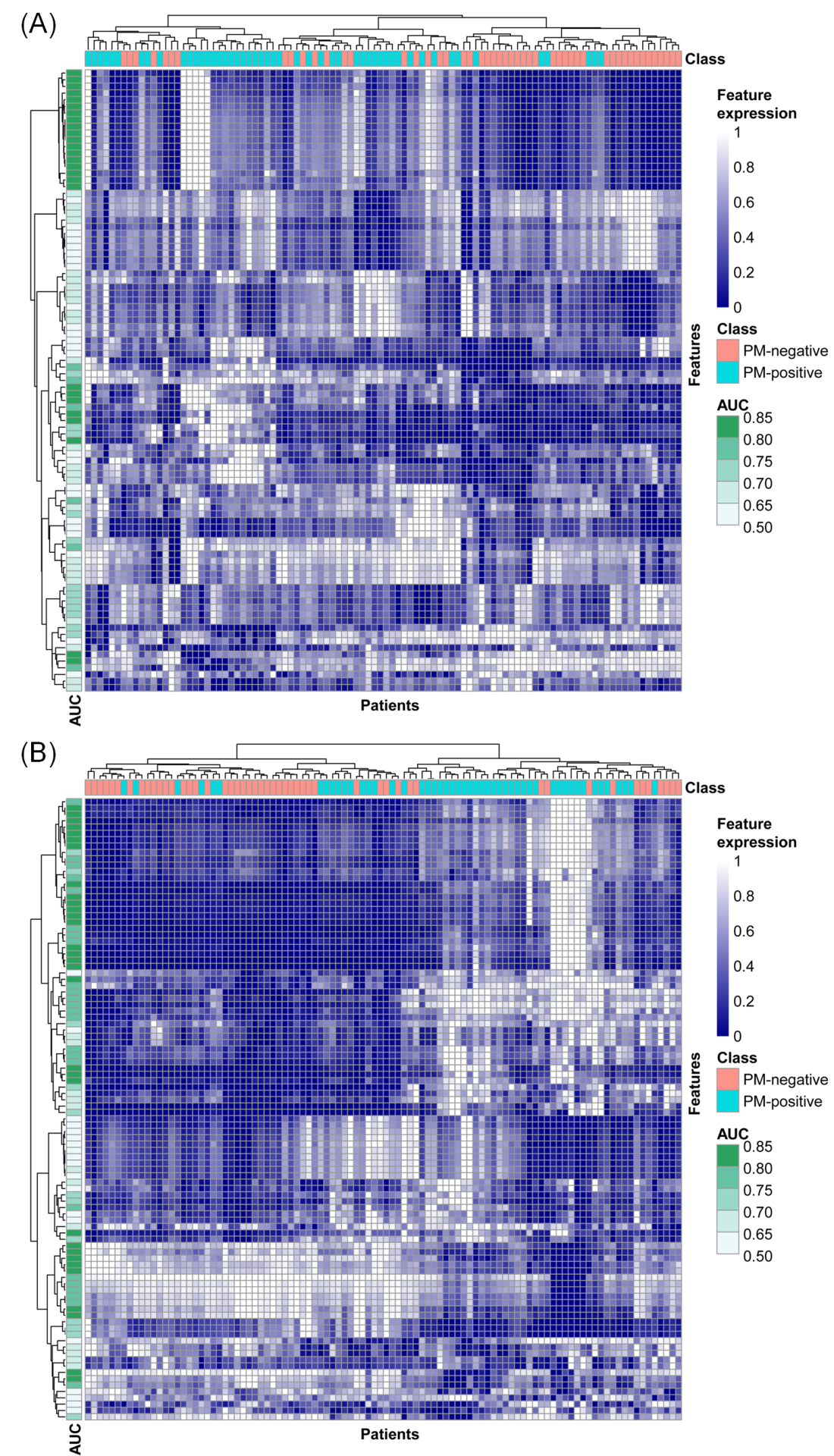


**Supplementary Figure S2.** Unsupervised cluster of patients on horizontal axis and radiomic features from primary tumor (A) and from peritoneal regions (B) on vertical axis. Unsupervised clustering with the Euclidean distance metric was used. To achieve a clear view, the feature expression has been normalized to the range 0-1. The peritoneal metastasis status of patients (PM-negative or PM-positive) and the AUC yielded by each feature (0.8-0.85, 0.75-0.8, 0.7-0.75, 0.65-0.7 or lower than 0.65) were exhibited as well.
