# Supplementary material for: Development and validation of an individualized nomogram to identify occult peritoneal metastasis in patients with advanced gastric cancer
Source: Ann Oncol. 2019 Jan 23;30(3):431–8. doi: 10.1093/annonc/mdz001 (PMC6442651; doi:10.1093/annonc/mdz001)
Supplement: Supplementary Data [file mdz001_supp.zip › mdz001-suppl_data/mdz001_Supplementary_Figure_S4.docx]

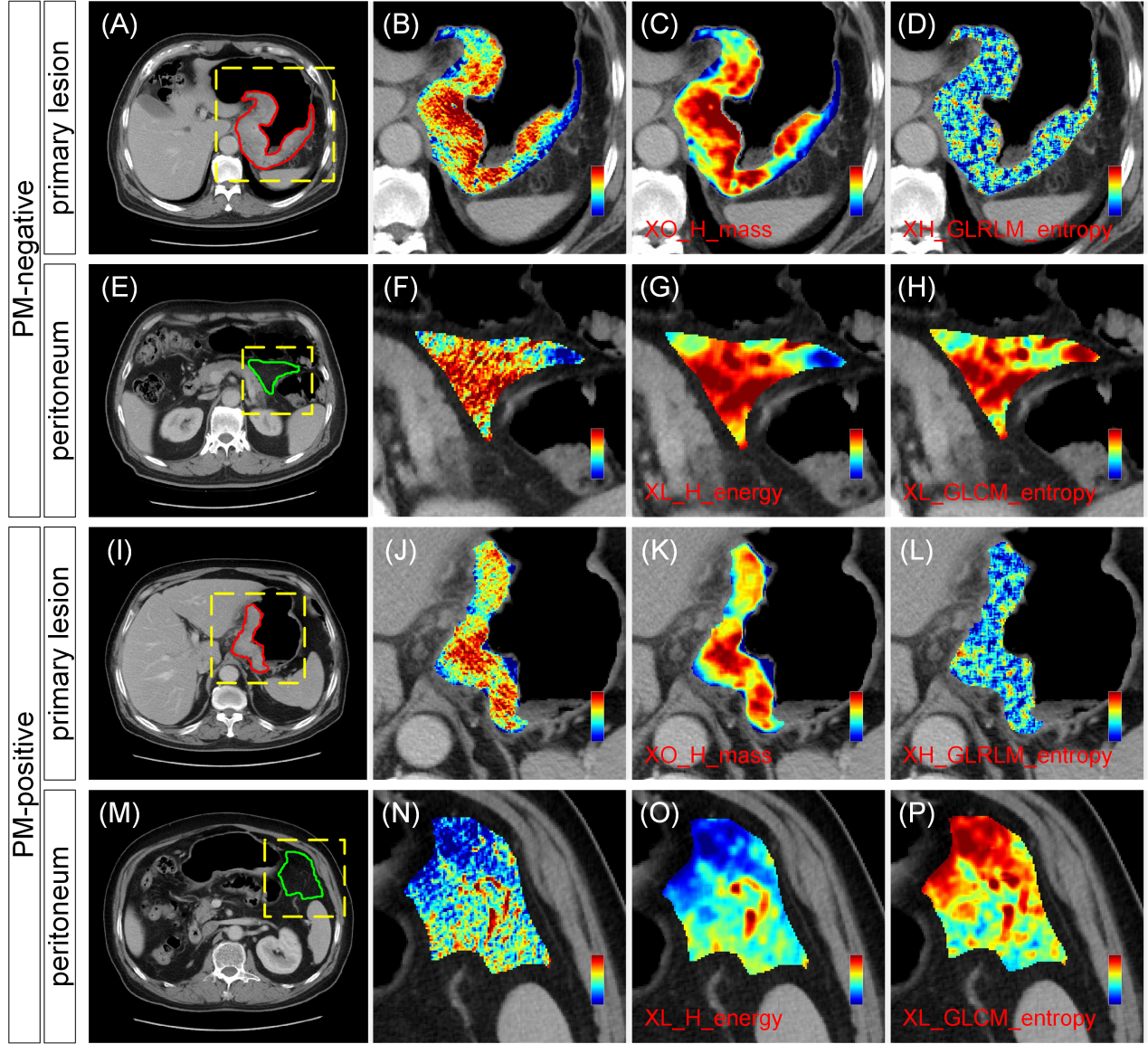


**Supplementary Figure S4.** Example of feature maps of a PM-negative patient (A-H; RS1 = 0.786; RS2 = 0.173; predicted risk of PM = 0.136) and a PM-positive patient (I-P; RS1 = 0.662; RS2 = 0.861; predicted risk of PM = 0.932). The columns show the primary tumor (A, I) overlaid by pseudo color of pixel intensity (B, J), the XO_H_mass (C, K) and the XH_GLRLM_entropy (D, L), and the peritoneum (E, M) overlaid by pseudo color of pixel intensity (F, N), the XL_H_energy (G, O) and XL_GLCM_entropy (H, P). The texture features were computed per voxel by using a 5 × 5 patch centered at each voxel. It should be noted that, to obtain a better exhibition, we selected a PM-negative patient with high RS1, as the primary tumor was larger than other PM-negative patients.
