# Supplementary material for: Development and validation of an individualized nomogram to identify occult peritoneal metastasis in patients with advanced gastric cancer
Source: Ann Oncol. 2019 Jan 23;30(3):431–8. doi: 10.1093/annonc/mdz001 (PMC6442651; doi:10.1093/annonc/mdz001)
Supplement: Supplementary Data [file mdz001_supp.zip › mdz001-suppl_data/mdz001_Supplementary_Figure_S7.docx]

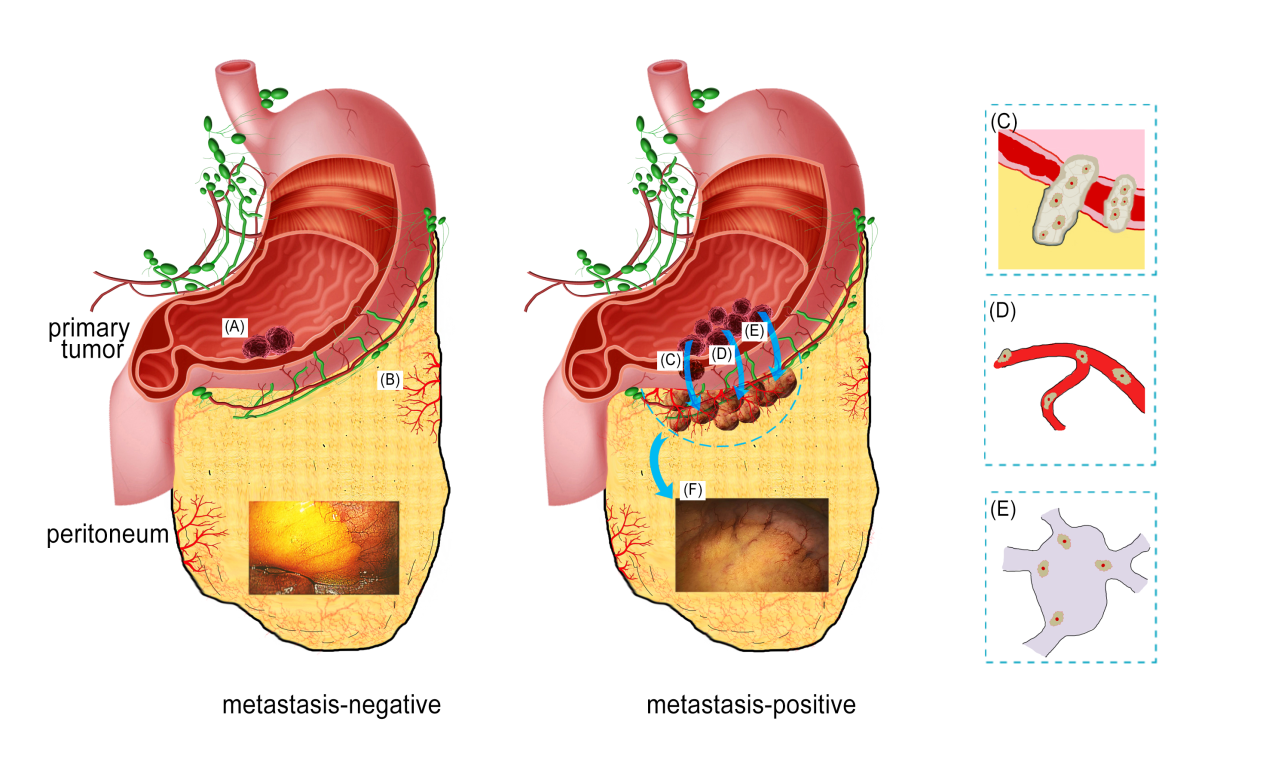


**Supplementary Figure S7**. Steps of the metastatic process. Metastasis is characterized by a series of sequential steps: primary tumor formation (A), recruitment of blood vessels through angiogenesis (B), cancer cell invasion of local tissue (C), and entry into dispersal corridors such as blood vessels (D) and lymph vessels (E). Disseminated cells travel through the circulation and upon reaching a suitable secondary site, extravasate from the blood vessels and colonize to form peritoneal metastasis (F).
