# Supplementary material for: Development and validation of an individualized nomogram to identify occult peritoneal metastasis in patients with advanced gastric cancer
Source: Ann Oncol. 2019 Jan 23;30(3):431–8. doi: 10.1093/annonc/mdz001 (PMC6442651; doi:10.1093/annonc/mdz001)
Supplement: Supplementary Data [file mdz001_supp.zip › mdz001-suppl_data/mdz001_Supplementary_Figure_S6.docx]

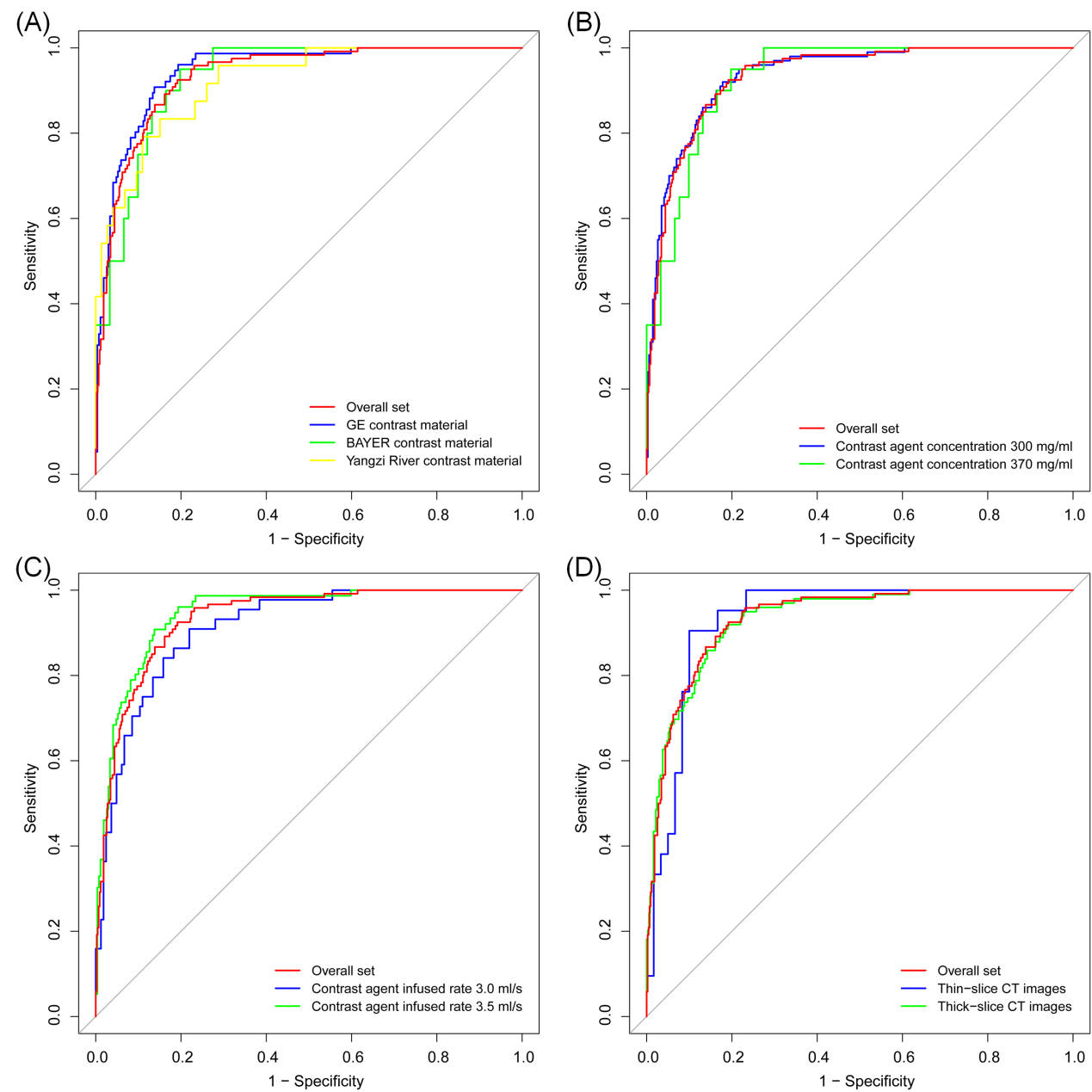


**Supplementary Figure S6**. Radiomic nomogram score for each subgroup stratified by (A) type of contrast agent, (B) contrast agent concentration, (C) contrast agent infused rate, and (D) image thickness.
