# Supplementary material for: Development and validation of an individualized nomogram to identify occult peritoneal metastasis in patients with advanced gastric cancer
Source: Ann Oncol. 2019 Jan 23;30(3):431–8. doi: 10.1093/annonc/mdz001 (PMC6442651; doi:10.1093/annonc/mdz001)
Supplement: Supplementary Data [file mdz001_supp.zip › mdz001-suppl_data/mdz001_Supplementary_Table_S3.docx]

**Table S3.** Description and univariate analysis of the selected radiomic features.

| Region | Features | Group | Filters | Univariate analysis  *p* value | Description |
| --- | --- | --- | --- | --- | --- |
| primary tumor | XO_H_mass | Histogram | NA | <0.0001 | It sums the intensity of the voxels and tends to emphasize the large tumor with high intensity level. |
|  | XH_GLRLM_entropy | GLRLM | *X_HH_* | <0.0001 | It computes the entropy of the GLRLM and tends to emphasize the regions with variable intensity patterns. |
| peritoneum | XL_H_energy | Histogram | *X_LL_* | <0.0001 | It computes the sum of the squares of the histogram and tends to emphasize the non-uniformity of intensity. |
|  | XL_GLCM_entropy | GLCM | *X_LL_* | <0.0001 | It computes the entropy of the GLCM and tends to emphasize the coherence of the co-occurrence pairs. |

Abbreviations: H, Histogram; GLRLM, gray-level run-length matrix; GLCM, gray-level co-occurrence matrix.
