# Supplementary material for: Development and validation of an individualized nomogram to identify occult peritoneal metastasis in patients with advanced gastric cancer
Source: Ann Oncol. 2019 Jan 23;30(3):431–8. doi: 10.1093/annonc/mdz001 (PMC6442651; doi:10.1093/annonc/mdz001)
Supplement: Supplementary Data [file mdz001_supp.zip › mdz001-suppl_data/mdz001_Supplementary_Appendix.docx]

**Supplementary material Online for**

**“Development and validation of an individualized nomogram to identify occult peritoneal metastasis in patients with advanced gastric cancer”**

**Supplementary A1: Patient Recruitment**

This retrospective study was a multi-center study with 326 patients from center 1 (Peking University Cancer Hospital & Institute in China), 111 patients from center 2 (The First Affiliated Hospital of Zhengzhou University in China), 20 patients from center 3 (Yunnan Cancer Hospital in China) and 97 patients from center 4 (Affiliated People’s Hospital of Jiangsu University in China). All patients were divided into four cohorts: a training cohort, an internal validation cohort, and two external validation cohorts. The training cohort contained 100 patients (50 PM-negative and 50 occult PM-positive) treated between September 2012 and October 2015 in center 1. The internal validation cohort contained 226 consecutive patients (206 PM-negative and 20 occult PM-positive) treated between November 2015 and December 2016 in center 1. The first external validation cohort included 131 patients (104 PM-negative and 27 occult PM-positive) from center 2 and center 3, and the second external validation cohort included 97 patients (73 PM-negative and 24 occult PM-positive) from center 4.

The inclusion and exclusion criteria were shown as follows.

The inclusion criteria were as follows: (1) diagnosed advanced gastric cancer (≥cT3) by endoscopy-biopsy pathology, combined with CT and/or endoscopic ultrasound; (2) with both enhanced CT and laparoscopy; (3) without typical PM indications in CT (diffuse omental nodules or omental cake, large amount of ascites, obvious irregular thickening with high peritoneal enhancement);^1^ (4) without other evidence of distant metastasis, and no stage IV features on CT.

The exclusion criteria were the following: (1) previous abdominal surgery; (2) previous abdominal malignancies or inflammatory diseases; (3) time intervals between CT and laparoscopy longer than 2 weeks; and (4) CT image artifacts that undermine peritoneal lesion assessment.

In center 1, AGC patients who underwent both contrast enhanced CT and laparoscopy between September 2012 and December 2016 were screened. 713 Patients meeting the inclusion/exclusion criteria (643 PM-negative and 70 PM-positive) were divided into a training cohort and an internal validation cohort taking October 2015 as time dividing point. There are 487 patients (50 occult PM-positive and 437 PM-negative) between September 2012 and October 2015 from center 1. All the 50 occult PM-positive patients between September 2012 and October 2015 were enrolled in training cohort. In order to balance the training samples, we randomly selected 50 PM-negative patients from 437 PM-negative patients between September 2012 and October 2015 from center 1 as the control group in the training cohort. While for the internal validation cohort, we intended to test our nomogram in a real experiment, with all 226 patients (206 PM-negative and 20 PM-positive) between November 2015 and December 2016 from center 1 being included.

In center 2, 111 patients between January 2012 and January 2018 met the inclusion/exclusion criteria with 91 PM-negative and 20 PM-positive.

In center 3, since laparoscopy was not regarded as a routine diagnosis, only 20 patients between September 2012 and December 2016 met the inclusion/exclusion criteria with 13 PM-negative and 7 PM-positive. The patients of center 2 and center 3 were all enrolled into the external validation cohort 1.

In center 4, 97 patients between December 2011 and March 2018 met the inclusion/exclusion criteria with 73 PM-negative and 24 PM-positive with several clinical characteristics investigated in this study lacked, e.g. Lauren type, Borrmann type. These patients were enrolled into the external validation cohort 2.

The flow diagram of study population is shown in Figure S1.

**Supplementary A2: Sample size consideration**

**Training cohort**

We retrospectively chose 100 consecutive patients treated in center 1 between September 2012 and October 2015 as training cohort. Considering that logistic regression performs best when case-to-noncase ratio is 1:1, we designed the training cohort to contain 50 occult PM-positive patients and 50 PM-negative patients who were randomly selected from the 437 PM-negative patient pool. There were in total 5 predictors in our nomogram, making an event-per-predictor ratio of 10. Vittinghoff and colleagues proposed that the rule of thumb for event-per-predictor in logistic regression models should be bigger than 5 to 9. ^2^ Therefore, we believed that there was no big concern on the overfitting issue of our model.

**Validation cohorts**

For the validation sample size, Shein-Chung Chow and colleagues^3^ introduced a sample size estimation method for clinical research. According to their book, the sample size calculation to test whether the means of two groups are significantly different refers to the following formula.

Letting the two groups be $A$ and $B$, $\mu$ represents the mean in each group, with the hypotheses of interest being:

$$H_{0}: \mu_{A}-\mu_{B}=0$$

$$H_{1}: \mu_{A}-\mu_{B}\neq0$$

The sample size and power are calculated respectively:

$$N_{A}=\left( \frac{n_{A}+n_{B}}{n_{B}} \right)\left( \sigma\frac{z_{1-\alpha/2}+z_{1-\beta}}{\mu_{A}-\mu_{B}} \right)^{2}$$

$$N_{B}=\left( \frac{n_{A}+n_{B}}{n_{A}} \right)\left( \sigma\frac{z_{1-\alpha/2}+z_{1-\beta}}{\mu_{A}-\mu_{B}} \right)^{2}$$

$1-\beta=\Phi\left( z-z_{1-\alpha/2} \right)+\Phi\left( -z-z_{1-\alpha/2} \right)$, $z=\frac{\mu_{A}-\mu_{B}}{\sigma\sqrt{\frac{1}{n_{A}}+\frac{1}{n_{B}}}}$

where, $n$ is the sample size in the training group and $N$ is the sample size for the validation group, $\Phi$ is the standard Normal distribution function, $\alpha$ is the Type I error, $\beta$ is the Type II error, $1-\beta$ is the power, and $\sigma^{2}$ is the variance of the covariate.

In our study, the sample sizes in the training groups were $n_{A}=50$ and $n_{B}=50$ with means of $\mu_{A}=0.1583$ and $\mu_{B}=0.8417$, respectively, and with a standard deviation of $\sigma=0.4169$. Therefore, the minimum number of validation samples were 10 (PM-negative) and 10 (PM-positive) in the two groups with the desired two-sided significance level of $\alpha$ = 0.05 and power of $1-\beta$ = 95%. In our study, the internal validation cohort included 206 PM-negative and 20 PM-positive patients in the two groups, respectively, which all exceeded the minimum required sample sizes. Besides, the two external validation cohorts included 104 PM-negative, 27 PM-positive patients and 73 PM-negative, 24 PM-positive patients respectively, which were also more than the minimum required sample sizes.

**Supplementary A3: CT examinations**

Enrolled patients in the four centers underwent similar scan setup but with different systems and parameters. All patients were overnight fasted and 20 mg of scopolamine or anisodamine were administered intramuscularly to reduce gastrointestinal peristalsis 15–20 min prior to CT examination. Moreover, patients drank 600–1000 ml warm water or received 6 g of effervescent granules to distend the stomach prior to CT examination.

All patients underwent contrast enhanced CT examinations in axial plane after injection of contrast agent with a pump injector into the antecubital vein. The CT scans, covering the entire stomach region, were acquired during a breath-hold with the patient supine. The CT image acquisition parameters of the four centers were shown in Table S1.

The diagnosis was performed with a standardized dynamic window adjustment procedure, on window-adjustable PACS work stations. Initially, a narrow window was used to demonstrate the primary tumor, and the window was then widened to observe the peritoneal status, which included the comprehensive region of the peritoneum and omentum. The optimal window for the detection of PM should clearly display the tiny grainy background noise of the fat tissue. Three-plane images (axial, coronal, and sagittal) were observed together to facilitate the detection and location of the PM.

**Supplementary A4: laparoscopy procedure**

Laparoscopy was performed under general anesthesia. The patients were placed in a supine position, and the operating table was repositioned according to the intra-abdominal region to be examined. A 10-mm disposable trocar was inserted into the sub-umbilicus, and the laparoscopy was performed using a 30° telescope. Another 10-mm trocar and a 5-mm trocar were inserted into the right and left upper quadrant, respectively. Prior to any manipulation, 250 mL of warm saline was infused into the subphrenic space, subhepatic space, omentum, bilateral paracolic sulci, and the pouch of Douglas. The irrigate was not administered over the primary tumor. After gentle agitation of the lower abdomen, 100 mL of fluid was aspirated from the subphrenic space, subhepatic space, and Douglas’ pouch. The ascites fluid was immediately sent for cytology examination including: 1) The ascites fluid was sent for centrifugation and the supernate was removed; 2) The residue sample was mounted on the slides and then fixed and stained; 3) The slides were finally put under a microscope to found if there is malignant cells by pathologists. Then, a systematic inspection of the abdominal cavity was performed clockwise from the right quadrant. After the patient was placed in the Trendelenburg position, the diaphragm, liver surface, parietal peritoneum, bilateral paracolic sulci, omentum, and the pouch of Douglas were inspected. Any suspicious lesion would be biopsied and sent for intraoperative frozen pathologic examination.

The whole procedure was recorded using a video recorder. The metastasis areas were located through the combination of intraoperative observation and postoperative video review.

**Supplementary A5: Radiomic feature extraction**

A filtering process was performed to implement image smoothing and image difference before CT radiomic feature extraction. Separable filtering was used to avoid the multi-dimensional convolution. The convolution was performed with a low-/high-pass “Coiflet 1” wavelet filter along the x-/y-direction, separately. Consider *L* and *H* to be low-pass and high-pass functions, respectively, *X* to be the original CT image, and the filtered results of *X* to be labelled as$X_{LL},X_{HH}$. That is, two new images were obtained by filtering the original image in two directions (x, y).

After filtering, a total of 133 quantitative features were extracted from the ROI of the original image and its corresponding filtered results, including the features from the categories of histogram, shape, gray-level co-occurrence matrix (GLCM), and gray-level run-length matrix (GLRLM). The process of image filtering and feature extraction was performed using an in-house software implemented in MATLAB (version 2017a; Mathworks, Natick, MA, USA).

Radiomic features of all patients were standardized by the z-score method, based on the parameters calculated from the training cohort.

**Supplementary A6: Radiomic feature selection and signature building process**

One radiologist with 10 years of experience with CT (reader 1) performed segmentations for all patients. Three months after the initial segmentation, 50 patients in the training cohort were randomly selected and re-segmented by this radiologist to assess intra-reader agreement in radiomic features. These cases were then segmented by another radiologist with 5 years of experience with CT (reader 2) for inter-reader agreement assessment. Meanwhile, a slice-thickness dataset was simulated based on the 3-dimensional images of 30 patients selected randomly to assess the robustness of features from slices with different thicknesses.

The feature selection and signature building process was performed on both primary tumor and peritoneum including three steps: 1) feature reproducibility assessment on inter/intra-reader agreement and slice-thickness agreement; 2) reservation of top ranking features; 3) signature building with three methods.

1) Based on the different independent segmentation groups, intra-/inter-class correlation coefficients (ICCs) were used to estimate each feature’s reproducibility. Stable features with ICCs >0.8 were reserved. Using the same strategy, the robustness of the features from slices with different thickness was also assessed using ICCs from the simulated slice-thickness data.

2) Features were ranked using the minimum redundancy maximum relevance (mRMR) algorithm by calculating the mutual information (MI) between features and PM status. As mentioned in the power calculation, the desired number of predictors should be no more than 1/3 of the smallest group in the training cohort (*n*=50). In this study, only 20 highest-ranking features in mRMR were reserved.

3) For radiomic signature building, the Least Absolute Shrinkage and Selection Operator Method (LASSO) logistic regression model, Support Vector Machine (SVM) using a Radial Basis Function (RBF) kernel, and a 3-layered, feed-forward Artificial Neural Network (ANN) were compared using 1,000 times 10-fold cross-validation in the training cohort, with the best performing model being selected.

After the three steps, a radiomic signature reflecting primary tumor (RS1) and another radiomic signature reflecting peritoneum microenvironment (RS2) were built as independent predictors for occult PM.

**Supplementary A7: Radiomics signatures calculation formula**

RS1 = Sigmoid[– 4.439 + 5.33 × (10^-6) × XO_H_mass + 7.445 × (10^-4) × XH_GLRLM_entropy]

RS2 = Sigmoid[– 6.183 + 8.848 × (10^-7) × XL_H_energy + 1.576 × (10^0) × XL_GLCM_entropy]

Sigmoid(x) = 1 / (1 + exp(– x))

Note:

XO_H_mass is calculated based on the original image. And the formula of mass is

$$mass=\sum_{i}^{N} X(i)$$

Where $X$ indicates the image with $N$ voxels.

XH_GLRLM_entropy is calculated based on the GLRLM of the image filtered directionally with high-pass filter along x and y directions. And the formula of entropy is

$$entropy=-\sum_{i=1}^{N_{g}} \sum_{j=1}^{N_{r}} p(i,j)\log_{2} p(i,j)$$

Where $P(i,j)$ is the $\left( i,j \right)$th entry in the GLRLM, $N_{g}$ the number of intensity values in the image, and $N_{r}$ the number of different run lengths.

XL_H_energy is calculated based on the image filtered directionally with low-pass filter along x and y directions. And the formula of energy is

$$energy=\sum_{i}^{N} {P(i)}^{2}$$

Where $X$ indicates the image with $N$ voxels.

XL_GLCM_entropy is calculated based on the GLCM of the image filtered directionally with low-pass filter along x and y directions. And the formula of entropy is

$$entropy=-\sum_{i=1}^{N_{g}} \sum_{j=1}^{N_{g}} P\left( i,j \right)\log_{2} \left[ P\left( i,j \right) \right]$$

where $P(i,j)$ be the co-occurrence matrix, $N_{g}$ be the number of discrete intensity levels in the image.

**Supplementary A8: Consistency of the radiomic signatures among slice selection by radiologists**

Considering that RS1 and RS2 were extracted from single CT slices, they might be affected by slice selection of the radiologists. Therefore, we tested the consistency of the radiomic signatures among the slice selection of ROI-1 and ROI-2 in one patient.

We randomly chose 30 patients from our cohort including 15 occult PM and 15 PM negative patients. For each patient, we asked the radiologist to select three slices near/include the largest-tumor-area slice per patient, and three primary tumor regions (ROI-1s) were segmented per patient. We calculated the ICC of RS1 scores in the three tumor regions per patient and found that RS1 scores had an ICC of 0.91 among the 30 patients, indicating that the consistency of RS1 was good. For the peritoneum, we asked the radiologists to select three slices per patient with all peritoneums nearby the primary tumor. Then, three peritoneal regions (ROI-2s) were segmented per patient. We calculated the ICC of radiomic signature in the three peritoneal regions per patient and found that RS2 scores had an ICC of 0.92 among the 30 patients, indicating that the consistency of RS2 was also very good.

In conclusion, the two signatures had a good consistency among the slice selection by radiologists.

**Supplementary A9: Stratified analysis of radiomic nomogram**

The generalizability of a nomogram is always of great concern in terms of its application values. BMI was believed to influence the performance because higher BMI patients have thicker peritoneum. Moreover, version of CT, type of contrast, and image thickness might also affect the CT phenotype. In order to test the generalization ability of our nomogram, we performed stratification analysis on the subgroups of sex, age, BMI, version of CT, type of CT contrast agent, contrast agent concentration, contrast agent infused rate and CT image thickness. We used the ROC curve and AUC to evaluate the performance of our nomogram on these subpopulations. The results showed that our nomogram was not influenced by these factors (all *p*>0.05) (see Supplementary Figure S5 and S6), indicating a good generalizability of the nomogram.

**1. Stratified analysis on age：**Patients are divided into two subgroups: age < 60 and age >= 60 with AUC of 0.928 and 0.936 (Delong test *p* value: 0.8026 and 0.8994 compared with the result on the overall cohort).

**2. Stratified analysis on sex：**Patients are divided into two subgroups: male and female with AUC of 0.927 and 0.944 (Delong test *p* value: 0.7336 and 0.6043 compared with the result on the overall cohort).

**3. Stratified analysis on patient BMI：**Patients are divided into two subgroups: BMI < 24 and BMI >= 24 with AUC of 0.948 and 0.937 (Delong test *p* value: 0.9271 and 0.7605 compared with the result on the overall cohort).

**4. Stratified analysis on version of CT system：**Patients are divided into two subgroups: scanned using GE CT system and using SIEMENS CT system with AUC of 0.941 and 0.966 (Delong test *p* value: 0.6201 and 0.1897 compared with the result on the overall cohort).

**5. Stratified analysis on type of contrast agent：**Patients are divided into three subgroups: infused the contrast agent of GE Healthcare, Bayer Schering Pharma and Yangzi River Pharmaceutical Group with AUC of 0.945, 0.930 and 0.920 (Delong test *p* value: 0.5007, 0.9048 and 0.6757 compared with the result on the overall cohort)

**6. Stratified analysis on contrast agent concentration:** Patients are divided into two subgroups: concentration = 300 mgI/ml and concentration = 370 mgI/ml with AUC of 0.936 and 0.930 (Delong test *p* value: 0.8591 and 0.9048 compared with the result on the overall cohort).

**7. Stratified analysis on contrast agent infused rate:** Patients are divided into two subgroups: infused rate = 3.0 ml/s and infused rate = 3.5 ml/s with AUC of 0.912 and 0.945 (Delong test *p* value: 0.3763 and 0.5007 compared with the result on the overall cohort).

**8. Stratified analysis on image thickness:** Patients are divided into two subgroups: image thickness = 1.25 or 2 mm and image thickness = 5 mm with AUC of 0.933 and 0.932 (Delong test *p* value: 0.9984 and 0.9090 compared with the result on the overall cohort).

**Supplementary A10: Benefit of radiomic nomogram on the internal validation cohort**

For the internal validation cohort, we aimed to quantify the benefit ratio of the radiomic nomogram in a real experiment which included with all patients meeting criteria between November 2015 and December 2016 in center 1. There were 20 out of 226 patients diagnosed as PM-positive through laparoscopy exploration who were diagnosed as PM-negative by CT staging. Therefore, if all study patients underwent laparoscopy exploration, 20 PM-positive patients would have been detected, but 206 patients will have received excessively invasive procedures. In addition, if no laparoscopy explorations were performed on this cohort, 20 patients would have the risk of an improper surgical procedure.

According to the NCCN guideline, clinicians are recommended to perform laparoscopy on all 226 patients to avoid the 20 improper surgical procedures. If we use our radiomic nomogram in the internal validation cohort, 44 patients would have been diagnosed as PM-positive, among whom 17 were true-positive; 182 patients would have been diagnosed as PM-negative, among whom 3 were false-negative; 44 patients would have underwent laparoscopy exploration, among whom only 27 will have unnecessary invasive exploration; 182 patients would not have undergone laparoscopy exploration, among whom only 3 would unnecessarily incur the risk of improper surgical procedures (planned laparoscopy resection could also have been administered, and the unnecessary risk would have been further reduced). The above results demonstrate the improved efficacy with the radiomic nomogram. Furthermore, the 3 patients misdiagnosed by the radiomic nomogram may undergo laparoscopy operation, have PM correctly identified, and thereafter avoid an improper surgical procedure, thereby avoiding further surgical trauma.

**References of the Supplementary:**

1. Kim S J, Kim H H, Kim Y H, et al. Peritoneal metastasis: detection with 16- or 64-detector row CT in patients undergoing surgery for gastric cancer. Radiology 2009; 253(2): 407-415.
2. Vittinghoff E, Mcculloch C E. Relaxing the rule of ten events per variable in logistic and Cox regression. American Journal of Epidemiology, 2007, 165(6):710.
3. Chow S, Shao J, Wang H. Sample size calculations in clinical research. 2nd Ed. Chapman &Hall//CRC Biostatistics Series 2008.
