# Supplementary material for: Development and validation of an individualized nomogram to identify occult peritoneal metastasis in patients with advanced gastric cancer
Source: Ann Oncol. 2019 Jan 23;30(3):431–8. doi: 10.1093/annonc/mdz001 (PMC6442651; doi:10.1093/annonc/mdz001)
Supplement: Supplementary Data [file mdz001_supp.zip › mdz001-suppl_data/mdz001_Supplementary_Table_S1.docx]

**Table S1.** The CT protocol of the four centers

| Parameters | Center 1  Peking University Cancer Hospital & Institute | Center 2  The First Affiliated Hospital of Zhengzhou University | Center 3  Yunnan Cancer Hospital | Center 4  Affiliated People’s Hospital of Jiangsu University |
| --- | --- | --- | --- | --- |
| CT version | Spectral CT (Discovery CT750 HD scanner, GE Healthcare, USA) | Spectral CT (Discovery CT750 HD scanner, GE Healthcare, USA) | 128-slice spiral CT (SOMATOM Deﬁnition AS+, Siemens Healthineers, Germany) | 256-slice spiral CT (Brilliance iCT, ROYAL PHILIPS, Netherlands)  or a 64-slice spiral CT (SOMATON sensation64, Siemens Healthineers, Germany) |
| CT  tube voltage | Spectral imaging mode switching between 120 kVp and 140 kVp | Spectral imaging mode switching between 120 kVp and 140 kVp | 120 kVp | 120 kVp |
| CT  tube current | 120-550 mA (the duration of automatically optimized to provide similar signal strength) | 375 mA | 290-330 mA | 220-250 mA |
| CT  rotation time | 0.76-0.80 s | 0.50-0.80 s | 0.50 s | 0.50-0.62 s |
| CT detector collimation | 64×0.625 mm | 64×0.625 mm | 128×0.6 mm | 128×0.625 mm or 32×0.6 mm |
| Contrast agent type | Omnipaque, GE Healthcare, USA | Ultravist 370, Bayer Schering Pharma, Germany | Omnipaque, GE Healthcare, USA | Iohexol, Yangzi River Pharmaceutical Group, China |
| Contrast agent concentration | 300 mgI/ml | 370mgI/ml | 300 mgI/ml | 300 mgI/ml |
| Contrast agent dosage | infused 1.5 ml/kg body weight | infused 1.5 ml/kg body weight | infused 1.5 ml/kg body weight | infused 1.5 ml/kg body weight |
| Contrast agent infused rate | 3.5 ml/s | 3.0 ml/s | 3.5 ml/s | 3.0 ml/s |
| Venous phase  interval time | 70 s after injection of  contrast agent | 70 s after injection of  contrast agent | 70 s after injection of  contrast agent | 70 s after injection of  contrast agent |
| Image matrix | 512×512 | 512×512 | 512×512 | 512×512 |
| Field of view | 500×500 mm | 500×500 mm | 500×500 mm | 500×500 mm |
| Reconstruction image thickness | 5 mm | 1.25mm or 5 mm | 2 mm | 5 mm |
