# Supplementary material for: Development and validation of an individualized nomogram to identify occult peritoneal metastasis in patients with advanced gastric cancer
Source: Ann Oncol. 2019 Jan 23;30(3):431–8. doi: 10.1093/annonc/mdz001 (PMC6442651; doi:10.1093/annonc/mdz001)
Supplement: Supplementary Data [file mdz001_supp.zip › mdz001-suppl_data/mdz001_Supplementary_Table_S2.docx]

**Table S2.** Cross-validation results of the models having highest AUCs built by each method.

| Method | primary tumor | | |  | peritoneum | | |
| --- | --- | --- | --- | --- | --- | --- | --- |
|  | Accuracy (95% CI) | Sensitivity | Specificity |  | Accuracy (95% CI) | Sensitivity | Specificity |
| RBF-SVM | 0.800 (0.709–0.885) | 0.822 | 0.778 |  | 0.821 (0.753–0.890) | 0.860 | 0.782 |
| ANN | 0.791 (0.690–0.923) | 0.828 | 0.754 |  | 0.807 (0.736–0.906) | 0.834 | 0.780 |
| LASSO-Logistic | 0.810 (0.746–0.881) | 0.845 | 0.775 |  | 0.842 (0.751–0.922) | 0.860 | 0.824 |

We used three methods to build the predictive signature through cross-validation on the training cohort. The Least Absolute Shrinkage and Selection Operator Method (LASSO) logistic regression model exhibited powerful predictive ability with accuracies of 0.810 (0.746–0.881) in primary tumor and 0.842 (0.751–0.922) in the peritoneal region respectively. SVM and ANN performed slightly worse in the cross-validation. With the optimal performance, the LASSO logistic regression model was therefore further used for radiomic signature building.

Abbreviations: RBF, radial basis function; SVM, support vector machine; ANN, artificial neural network; LASSO, least absolute shrinkage and selection operator; CI, confidence interval.
